# Delocalized excitons and interaction effects in extremely dilute thermal ensembles

**DOI:** 10.1039/c8cp05851b

**Published:** 2018-11-16

**Authors:** Lukas Bruder, Alexander Eisfeld, Ulrich Bangert, Marcel Binz, Max Jakob, Daniel Uhl, Markus Schulz-Weiling, Edward R. Grant, Frank Stienkemeier

**Affiliations:** a Institute of Physics , University of Freiburg , Hermann-Herder-Str. 3 , 79104 Freiburg , Germany . Email: lukas.bruder@physik.uni-freiburg.de; b Max Planck Institute for the Physics of Complex Systems , Nöthnitzer Strasse 38 , 01187 Dresden , Germany; c Department of Chemistry , University of British Columbia , Vancouver British Columbia , V6T 1Z1, Canada; d Freiburg Institute of Advanced Studies (FRIAS) , University of Freiburg , Albertstr. 19 , D-79194 Freiburg , Germany

## Abstract

Long-range interparticle interactions are revealed in extremely dilute thermal atomic ensembles using highly sensitive nonlinear femtosecond spectroscopy. Delocalized excitons are detected in the atomic systems at particle densities where the mean interatomic distance (>10 μm) is much greater than the laser wavelength and multi-particle coherences should destructively interfere over the ensemble average. With a combined experimental and theoretical analysis, we identify an effective interaction mechanism, presumably of dipolar nature, as the origin of the excitonic signals. Our study implies that even in highly-dilute thermal atom ensembles, significant transition dipole–dipole interaction networks may form that require advanced modeling beyond the nearest neighbor approximation to quantitatively capture the details of their many-body properties.

## Abstract 2

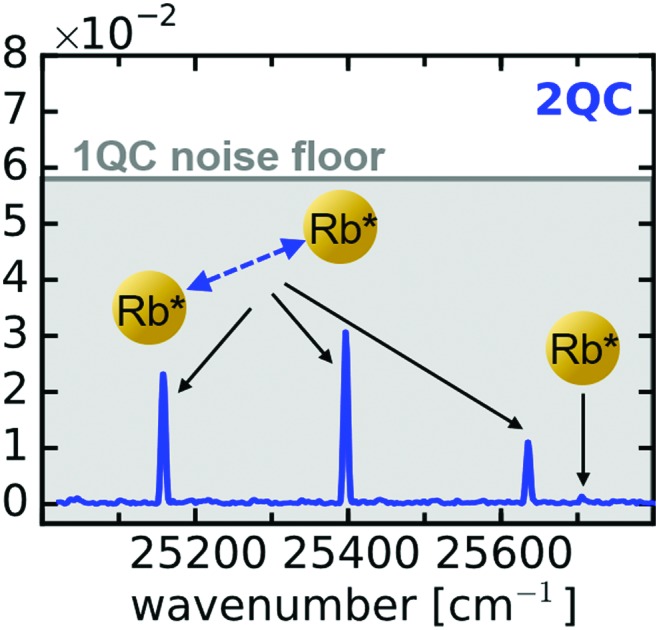
Long-range interparticle interactions are revealed in extremely dilute thermal atomic ensembles using highly sensitive nonlinear femtosecond spectroscopy.

## 1 Introduction

Dipolar interactions are the driving forces of most many-body quantum phenomena. Prominent examples range from magnetism, the formation of molecules^1^,^2^ to solvation mechanisms,^3^ the Rydberg blockade,^4^,^5^ interatomic coulombic decay^6^,^7^ and energy transport in biological complexes.^8^–^11^ Moreover, schemes have been proposed to engineer dipole interactions for quantum information processing^4^,^12^,^13^ and new states of matter.^14^ A full understanding of dipolar interactions is thus of immense interest. However, to rigorously test the current level of theory, an extension of studies to new systems and, in particular, to extreme regions of phase space is essential.

Dipole–dipole interactions have been extensively studied in ultra cold systems,^5^,^15^–^21^ where phase space is strongly confined and precision measurements can reveal even subtle interaction effects. On the contrary, thermal vapors cover a significantly larger parameter space and provide a more realistic approximation of natural systems, due to the thermal energy and motion of particles. Yet, the latter property causes inhomogeneous broadening (self-, Doppler broadening) dominating the system and requiring specialized nonlinear optics methods to uncover collective dipole interactions.^22^ By applying such schemes, many-body effects have been observed in dilute, thermalized Rydberg ensembles^23^–^25^ but also for excitation of lower principle quantum numbers (D line excitations) in alkali atom vapors.^26^–^31^

A particularly intriguing and unique feature of the dipole potential is its long-range nature, scaling with 1/*r*^3^ (*r* denotes the interparticle distance), due to which it was suggested that cooperative effects may be present at all particle densities.^22^ In the current work, we show a strong indication for this statement by applying a highly sensitive nonlinear spectroscopy method that allows us to study the long-range behavior of transition dipole–dipole interactions at extreme dilute conditions, demonstrated for the D line excitations in a thermal atomic rubidium (Rb) ensemble. Surprisingly, we find indications for a significant interaction among the atoms even at mean interparticle distances ( In the current work, we show a strong indication for this statement by applying a highly sensitive nonlinear spectroscopy method that allows us to study the long-range behavior of transition dipole–dipole interactions at extreme dilute conditions, demonstrated for the D line excitations in a thermal atomic rubidium (Rb) ensemble. Surprisingly, we find indications for a significant interaction among the atoms even at mean interparticle distances (〈*r*〉 > 10 μm) much greater than the wavelength of the coherent excitation field (∼790 nm) and at densities of ≲10 > 10 μm) much greater than the wavelength of the coherent excitation field (∼790 nm) and at densities of ⪅10^7^ cm^–3^, being five orders of magnitude smaller then so far reported in atomic vapors^32^ and about three orders of magnitude smaller than typical densities in ultracold atom clouds.^15^ Our results cannot be quantitatively reproduced by a two-body model, indicating, that the nearest-neighbor approximation is inappropriate in a non-ordered gas, even at the investigated ultra low densities.

## 2 Experimental method

To study weak interactions in a thermal ensemble of particles, time-domain spectroscopy methods are of advantage, as they effectively capture a snapshot of the system within a short period of observation time (frozen gas limit). Femtosecond (fs) time-domain spectroscopy has enabled the detection of transition dipole–dipole interactions down to vapor densities of ∼10^15^ cm^–3^ (ref. 29–31) and recently, with the development of a specialized coherent multidimensional spectroscopy method even down to 10^12^ cm^–3^.^32^–^34^ The latter technique has been also successfully applied to investigate bi- and higher order excitons in semiconductor nanostructures.^35^–^37^ This method relies on detecting the fs time evolution of multiple quantum coherences (MQCs) which correspond to electronic coherences simultaneously induced among multiple particles. It has been shown theoretically, that in a properly designed measurement protocol, these signals provide a unique, background-free probe of interparticle interactions,^38^ constituting the high sensitivity of the MQC-detection scheme.

**Fig. 1:**
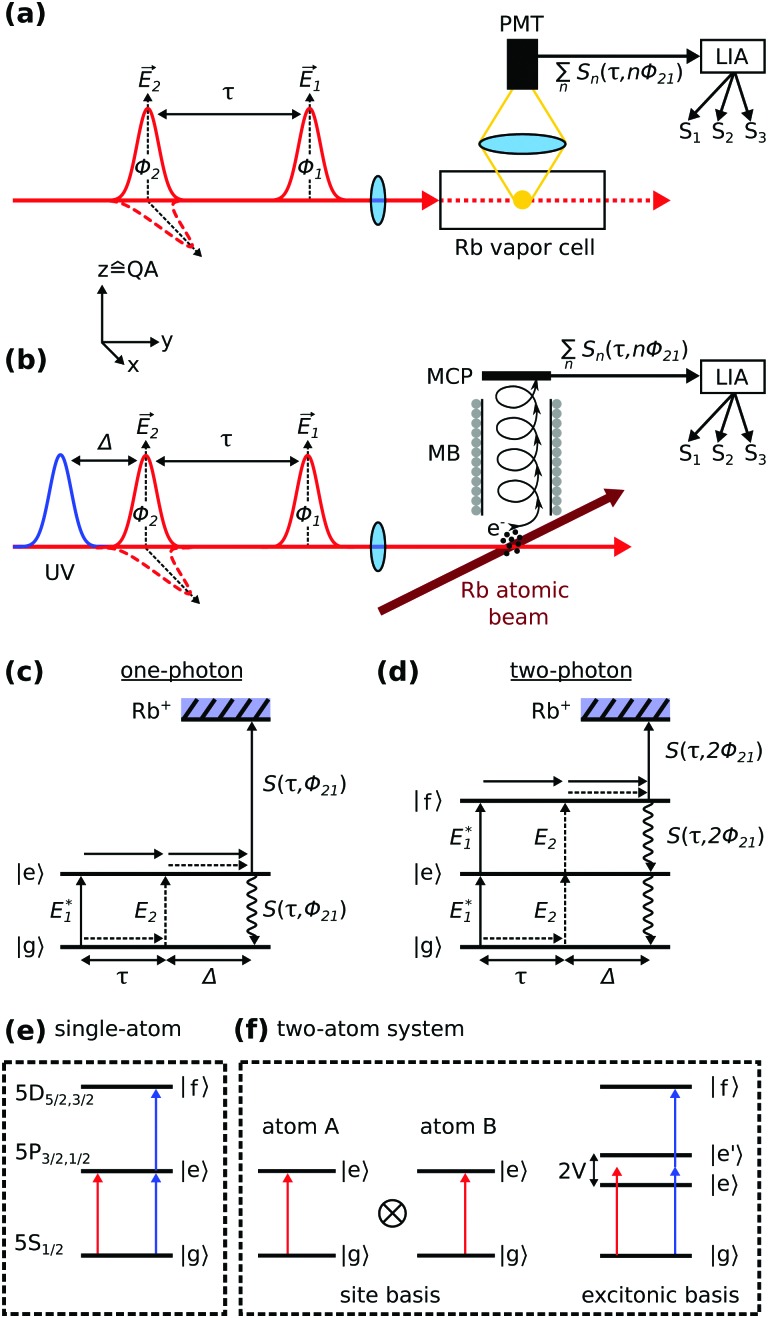
Experimental scheme. (a) Quantum beat fluorescence measurement in a Rb vapor. The fluorescence is detected with a photo multiplier tube (PMT) and the signal *S*_*n*_(*τ*,*nφ*_21_) is amplified and fed into a lock-in amplifier (LIA), where the phase modulation *φ*_21_(*t*) is removed from the signal and *S*_*n*_ is decomposed into its harmonic components *S*_*k*_, *k* = 1–3. Optionally, the probe pulse is orthogonally polarized to the pump pulse (indicated by dashed pulse envelope), where QA denotes the quantization axis of the system. (b) Quantum beat photoionization measurement of a thermal atomic Rb beam in a vacuum apparatus using a separate UV pulse (260 nm) for ionization. Photoelectrons are detected in a magnetic bottle (MB) spectrometer with a multi-channel plate (MCP) detector. (c and d) Interfering quantum pathways excited in the Rb atoms for one- and two-photon excitations, respectively. (e) Schematic representation of a single-atom energy structure comprised of three electronic states, along with one- (red) and two-photon excitations (blue). The states |g = 1–3. Optionally, the probe pulse is orthogonally polarized to the pump pulse (indicated by dashed pulse envelope), where QA denotes the quantization axis of the system. (b) Quantum beat photoionization measurement of a thermal atomic Rb beam in a vacuum apparatus using a separate UV pulse (260 nm) for ionization. Photoelectrons are detected in a magnetic bottle (MB) spectrometer with a multi-channel plate (MCP) detector. (c and d) Interfering quantum pathways excited in the Rb atoms for one- and two-photon excitations, respectively. (e) Schematic representation of a single-atom energy structure comprised of three electronic states, along with one- (red) and two-photon excitations (blue). The states |g〉 to |f〉 represent the individual energy states of the Rb atoms. (f) Two atoms, represented as two-level systems, described in the site- and excitonic basis. In the latter, an interatomic interaction to |f = 1–3. Optionally, the probe pulse is orthogonally polarized to the pump pulse (indicated by dashed pulse envelope), where QA denotes the quantization axis of the system. (b) Quantum beat photoionization measurement of a thermal atomic Rb beam in a vacuum apparatus using a separate UV pulse (260 nm) for ionization. Photoelectrons are detected in a magnetic bottle (MB) spectrometer with a multi-channel plate (MCP) detector. (c and d) Interfering quantum pathways excited in the Rb atoms for one- and two-photon excitations, respectively. (e) Schematic representation of a single-atom energy structure comprised of three electronic states, along with one- (red) and two-photon excitations (blue). The states |g〉 to |f〉 represent the individual energy states of the Rb atoms. (f) Two atoms, represented as two-level systems, described in the site- and excitonic basis. In the latter, an interatomic interaction represent the individual energy states of the Rb atoms. (f) Two atoms, represented as two-level systems, described in the site- and excitonic basis. In the latter, an interatomic interaction *V* lifts the degeneracy of the singly excited states. Note, that here the states |g lifts the degeneracy of the singly excited states. Note, that here the states |g〉 to |f〉 denote two-body states. to |f lifts the degeneracy of the singly excited states. Note, that here the states |g〉 to |f〉 denote two-body states. denote two-body states.

Recently, we have reported a simplified one-dimensional variant of this method with highly improved sensitivity.^39^ Its basic concept relies on a quantum beat experiment. The sample (ensemble of Rb atoms) is excited with two time-delayed fs pulses (Fig. 1a and b). The first pulse (pump) triggers a coherent time evolution of the system which is then converted into a population by the second pulse (probe, Fig. 1c). Thereby, the temporal delay *τ* and relative phase *φ*_21_ between the pulses causes a phase difference among the different quantum pathways evolving either on the ground or excited state of the system. This gives rise to constructive and destructive interference patterns (quantum beats) in the detected signal^40^ as in the fashion of a Young's double slit experiment.

**Fig. 2:**
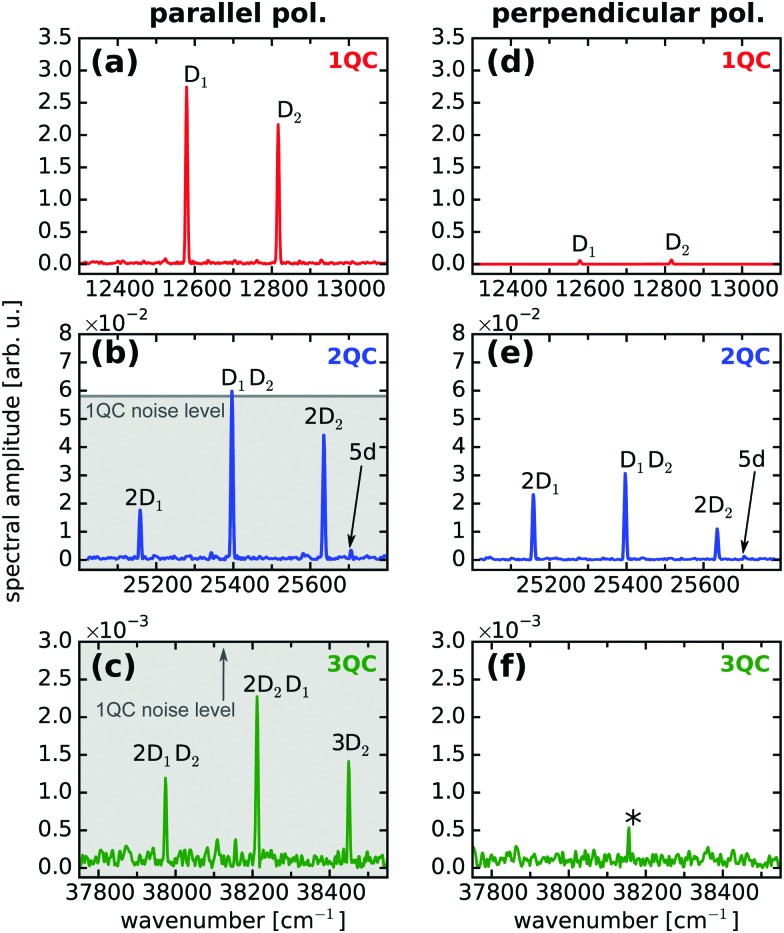
Fourier spectra of quantum beat measurements in a Rb vapor of moderate density (7 × 10^10^ cm^–3^). (a–c) 1QC–3QC data for parallel and (d–f) for perpendicular pump–probe polarization, respectively. D_1,2_ denote the D line excitations from the Rb ground state 5S_1/2_ to the 5P_1/2,3/2_ states and 5d the excitation to the 5D_5/2_ state, respectively. The combinations *m*D_1,2_*n*D_1,2_, *n*, *m* = 0, 1, 2 denote the collective two- and three-atom excitations. Low-frequency noise appearing at the lock-in amplifier reference frequency is marked with an asterisk. The noise floor level of the 1QC measurement is indicated as grey background in the 2QC and 3QC data to visualize the S/N advantage of the detection scheme.

More specifically, the phases *φ*_1,2_ of the electric fields *E*_1_ and *E*_2_ are imprinted on the pathways during excitation, leading to an interference signal with respect to *φ*_21_ = *φ*_2_ – *φ*_1_ for a one-photon excitation pathway (Fig. 1c) and 2*φ*_21_ for a two-photon excitation pathway (Fig. 1d), respectively. Likewise, the pathways accumulate different phase factors as a function of the pulse delay *τ* as they propagate on an excited or ground state, respectively. The relative accumulated phase is given by *φ*_*τ*_ = *ω*_eg_*τ* (*ω*_eg_ denotes the transition frequency between |g denotes the transition frequency between |g〉 and |e〉), and basically reflects the time evolution (including dephasing) of an electronic coherence prepared in the system by the pump pulse. Thereby, we distinguish coherences among electronic states separated by a one-photon energy gap [named one-quantum coherence (1QC)] or a and |e denotes the transition frequency between |g〉 and |e〉), and basically reflects the time evolution (including dephasing) of an electronic coherence prepared in the system by the pump pulse. Thereby, we distinguish coherences among electronic states separated by a one-photon energy gap [named one-quantum coherence (1QC)] or a ), and basically reflects the time evolution (including dephasing) of an electronic coherence prepared in the system by the pump pulse. Thereby, we distinguish coherences among electronic states separated by a one-photon energy gap [named one-quantum coherence (1QC)] or a *n*-photon gap [*n*-quantum coherence (*n*QC), *n* ∈ ℕ]. Accordingly, the *n*QC signal is expressed as

1 *S*_*n*_(*τ*,*nφ*_21_) ∝ cos(*ω*_*n*g_*τ* + *nφ*_21_)exp(–*γ*_*n*_*τ*),

where *γ*_*n*_ denotes the dephasing rate of the excited *n*-quantum coherence.

In the experiment, we induce a modulation [denoted *φ*_21_(*t*)] of the relative phase between pump and probe pulses which allows us to isolate the 1QC to *n*QC signals with a specialized lock-in detection scheme.^39^ Briefly, *φ*_21_ is modulated on a shot-to-shot basis to induce a beat note of kHz-frequency (*φ*_21_(*t*) = *Ωt*, *Ω* = 5 kHz) as parametric function of *τ* (*cf.*eqn (1)). This signal modulation is used for lock-in detection (Fig. 1a and b) which significantly improves the signal quality due to a passive stabilization effect as discussed in ref. 41 and 42. In addition, one- and *n*-photon excitation processes are modulated on respective harmonics of the *φ*_21_(*t*)-beat note and we can use harmonic lock-in detection to efficiently separate these signals from each other. At the same time, *τ* is scanned in discrete steps to monitor the time evolution of induced quantum coherences. A Fourier transform of the isolated quantum beat signals with respect to *τ* yields then the frequency response of the system, readily separated in the 1QC to *n*QC detection channels.

The quantum beat measurements are performed in a thermal Rb vapor combined with fluorescence detection (Fig. 1a) and in an effusive Rb atom beam prepared in a vacuum apparatus, combined with photoelectron detection (Fig. 1b). The atom density in the vapor cell is estimated from the temperature of the coldest spot (cold finger).^43^ The effusive atom beam is generated by evaporating rubidium in a heated reservoir placed inside the vacuum apparatus, described previously.^42^ The atom vapor expands through a reservoir opening of 3 mm diameter at low stagnation pressure, resulting in an effusive atom beam. The beam travels through the differentially pumped vacuum apparatus into the detector chamber, where the base pressure is <2 × 10^–9^ mbar. To calculate the atom beam density in the interaction volume, the procedure in ref. 44 is followed.

The laser beam is focused with a focal length of *f* = 500 mm (vapor cell) or *f* = 200 mm (magnetic bottle) into the interaction volume. The employed pump and probe laser pulses have a central wavelength of *λ*_L_ = 788 nm and Δ*λ*_FWHM_ ≈ 24 nm spectral width to cover both D line resonances of rubidium simultaneously and a separate ultra violet (UV) pulse at *λ* = 260 nm is used for photoionization. Pulse energies are ∼30 nJ (pump, probe) and 0.5 μJ (ionization pulse) and a laser repetition rate of 200 kHz is used. The probe pulse polarization is optionally rotated by 90° with respect to the pump pulse polarization using a zero-order *λ*/2-waveplate. The correct retardation of the waveplate over the whole pulse spectrum is confirmed with a Fourier analysis of cross-correlation measurements.

## 3 Results

Fig. 2a–c show the (Fourier-transformed) 1QC–3QC signals obtained for the Rb vapor (density of 7 × 10^10^ cm^–3^) using equal linear polarization for pump and probe pulses. For a single Rb atom, one- and two-photon transitions can be excited for the 5S_1/2_ → 5P_3/2,1/2_ and 5S_1/2_ → 5D_5/2,3/2_ resonances, respectively (schematically shown in Fig. 1e) which are detected as 1QC and 2QC signals in Fig. 2a and b (labeled D_1_, D_2_ and 5d).

The combination of multiple atoms (discussed here for the case of two Rb atoms) leads to an excitonic energy structure which as well supports multi-photon excitations (Fig. 1f). For simplicity, the atoms are approximated by two-level systems, however, the considerations are equivalent if including any substructure of states or higher-lying states. The respective resonances to the higher-lying collective energy states, *e.g.* two or three atoms simultaneously excited *via* the D_1,2_ lines or mixtures thereof, are remarkably well resolved in the experiment, despite the low atom density (Fig. 2b). Note, that we also observe 3QC signals corresponding to the combination of three atoms (Fig. 2c). These observations are in accordance with our previous study revealing two to four-atom collective resonances in a potassium vapor at similar densities (∼10^10^ cm^–3^).^39^ The *n*QC signals can be thus used as a sensitive probe to reveal many-body states, *i.e.* delocalized excitons, in the system.

Yet, in order to understand the nature of these many-body signals, the effect of interparticle interactions has to be discussed. Possible interaction mechanisms are transition dipole–dipole^5^,^15^,^24^ or van der Waals-type induced-dipole interactions.^12^,^21^,^23^,^45^ At the studied low atom densities and excitation of low principle quantum numbers, van der Waals interactions can be neglected. Likewise, electronic quadrupole or magnetic dipole transitions exhibit orders of magnitude smaller probabilities^46^ and are not observed in our experiment. As such, only the long-range transition dipole–dipole interaction should play a role in the experiments and we therefore focus on this effect in our work.

In a recent theoretical work, we have shown for a two-atom model system that a collective signal only appears in the quantum beat experiments if an interaction (here dipolar coupling) among the atoms is introduced.^47^ The underlying argumentation is equivalent to previous two-dimensional 2QC experiments,^32^,^38^ showing the similarity between the two- and one-dimensional nonlinear experiments. However, while the two-dimensional spectroscopy studies did not provide sufficient sensitivity to probe highly dilute samples (*i.e.* particle densities <10^12^ cm^–3^), our approach revealed for the first time collective resonances at densities where interactions are expected to be negligibly small (*V*/*h* ∼ Hz), thus raising general doubts about the interpretation of the MQC signals as a unique probe for interparticle interactions.^47^,^48^

In the current work, we resolve this issue by introducing a distinct variation of our experimental scheme which clearly eliminates all ambiguities and provides a direct indication for long-range interactions in the system. To this end, we additionally perform the quantum beat experiments using perpendicularly, linearly polarized pump–probe pulse sequences (Fig. 2d–f).

To discuss the effect of perpendicular pump–probe polarization, it is convenient to define the system's quantization axis parallel to the pump pulse polarization vector *Ê*_1_ (Fig. 1a and b). This yields for the *m*-level selection rules of the light-matter interactions Δ*M* = 0 (pump) and Δ*M* = ±1 (probe), respectively (*M* denotes the total magnetic quantum number of the electronic wavefunction). Consequently, the interference of one-photon excitation pathways vanishes in the quantum beat experiment as their magnetic states are orthogonal to each other (Fig. 1c). This leads to a depletion of the 1QC single-atom signals, in accordance with our experimental observation (Fig. 2d).

In contrast, for a two-photon excitation (Fig. 1d), the selection rules are Δ*M* = 0 (pump) and Δ*M* = ±2, 0 (probe). Hence, two-quantum pathways involving Δ*M* = ±2 transitions will not generate a detectable interference, whereas a fraction of the pathways (Δ*M* = 0 for pump and probe) will generate a signal even for perpendicular pump–probe polarization.^49^ This explains the residual signal in Fig. 2e, where we obtain similar signal amplitudes as for the parallel laser polarization (Fig. 2b) but certainly not a drastic amplitude drop as in case of the 1QC signals. Similar arguments apply for the 3QC signal, which again vanishes for perpendicular pump–probe polarization (Fig. 2f) in accordance with the Δ*M*-selection rules.

Note, that for perpendicular laser polarization a residual 1QC signal (2% of initial amplitude) is still observable in Fig. 2d due to a small fraction of parallel laser polarization remaining after the rotation of the probe beam. For confirmation, the optical interference of pump and probe electric fields was recorded for parallel and perpendicular polarization simultaneously to the quantum beat measurements (not shown), showing also a residual signal of 2% for the perpendicular polarization.

The signal drop in the 1QC signal is in clear contrast to the behavior of the 2QC signal, where we observe amplitude variations of up to a factor of two (Fig. 2b and e). Interestingly, the individual 2QC resonances exhibit different shifts in amplitude when going from parallel to perpendicular polarization. This might be explained by the different *m*-level substructures contributing to the individual two-atom configurations in combination with their possible orientations relative to the laser polarizations.

The general behavior for parallel and perpendicular pump–probe polarizations indicates the presence of interparticle interactions in the system as can be readily rationalized considering the model system given in Fig. 1f. An extension of our argumentation to more complicated level structures and larger numbers of particles is straight forward.

For absent interactions among the particles (*V* = 0), atom A and atom B (site representation) are independent, uncoupled particles, each supporting only one-photon excitations, but, in principle, the collective excitation of both atoms is possible. The latter would correspond to a two-photon transition (|e = 0), atom A and atom B (site representation) are independent, uncoupled particles, each supporting only one-photon excitations, but, in principle, the collective excitation of both atoms is possible. The latter would correspond to a two-photon transition (|e〉 → |f〉) in the excitonic description. However, for perpendicular laser polarization, the interference of one-photon excitation pathways clearly vanishes and hence no quantum beat signal can be generated in the single, uncoupled atoms described in the system's site representation. Since for absent interactions, site- and excitonic descriptions are in general equivalent, one can directly conclude, that neither 1QC nor 2QC signals are allowed for the combination of → |f = 0), atom A and atom B (site representation) are independent, uncoupled particles, each supporting only one-photon excitations, but, in principle, the collective excitation of both atoms is possible. The latter would correspond to a two-photon transition (|e〉 → |f〉) in the excitonic description. However, for perpendicular laser polarization, the interference of one-photon excitation pathways clearly vanishes and hence no quantum beat signal can be generated in the single, uncoupled atoms described in the system's site representation. Since for absent interactions, site- and excitonic descriptions are in general equivalent, one can directly conclude, that neither 1QC nor 2QC signals are allowed for the combination of ) in the excitonic description. However, for perpendicular laser polarization, the interference of one-photon excitation pathways clearly vanishes and hence no quantum beat signal can be generated in the single, uncoupled atoms described in the system's site representation. Since for absent interactions, site- and excitonic descriptions are in general equivalent, one can directly conclude, that neither 1QC nor 2QC signals are allowed for the combination of *V* = 0 and perpendicular laser polarization. The clear signature of 2QC signals in our data for perpendicular laser polarization (Fig. 2e) are in contrast to this and hence directly indicate the presence of an interparticle interaction in the system.

**Fig. 3:**
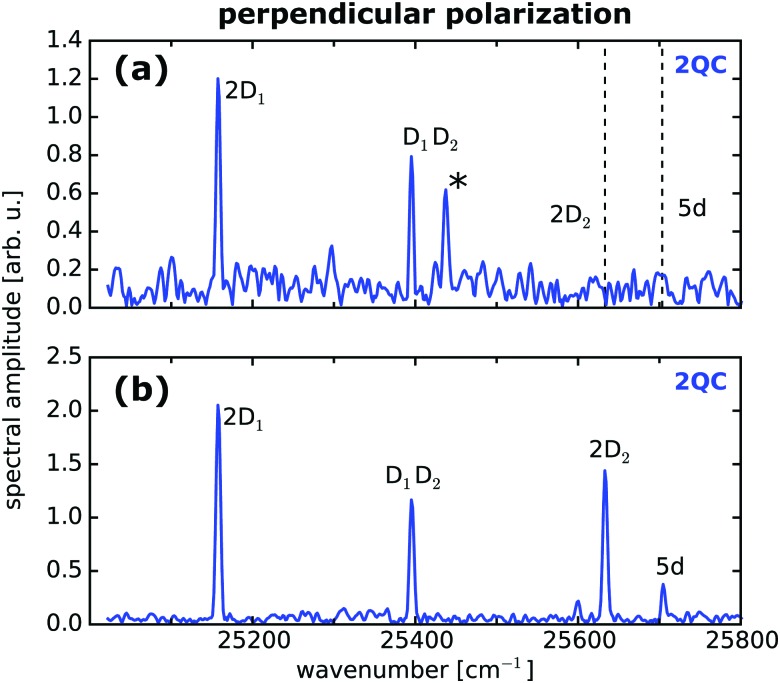
Two-atom interactions at low particle densities isolated with perpendicular pump–probe laser polarization. (a) Fluorescence-detected collective two-atom signals in a Rb vapor of density 8 × 10^9^ cm^–3^ and (b) for photoelectron-detection in a thermal Rb atom beam (density 8 × 10^6^ cm^–3^) produced in an ultra high vacuum environment. Labels are in accordance with Fig. 2. Dashed lines indicate the position of peaks covered by the noise floor.

Having identified the 2QC signal measured with orthogonally polarized pulses as a unique probe for interactions, we focused in the next step on more dilute samples while optimizing the sensitivity of our setup. As indicated in Fig. 2, the separate detection of 1QC–3QC signals in different lock-in detection channels provides a significant signal-to-noise (S/N) advantage as the higher-order detection channels provide greater discrimination of background signals that would otherwise cover the weak collective 2QC and 3QC signals. This allows us to reveal two-atom interactions at even lower vapor densities. As such, Fig. 3a shows a fluorescence-detected 2QC measurement of the Rb vapor at a particle density of 8 × 10^9^ cm^–3^.

To push the sensitivity of our experiment even further, we produced a highly dilute thermal atomic beam in an ultra-high vacuum environment and combined the quantum beat measurements with photoionization using a magnetic bottle spectrometer for monitoring the photoelectron yield of ionized Rb atoms (Fig. 1b). Photoionization has the advantage that charged particles can be more efficiently detected than photons. In particular, the employed detector provides an acceptance solid angle of nearly 4π for ejected electrons, thus improving the detection efficiency considerably compared to fluorescence measurements.

This is reflected in the improved signal quality (Fig. 3b) of 2QC resonances as compared to the fluorescence measurement (Fig. 3a). In the photoelectron measurement, the atom beam exhibits a particle density at the interaction region of the detector of only 8 × 10^6^ cm^–3^ which corresponds to a mean interatomic distance of which corresponds to a mean interatomic distance of 〈*r*〉 = 27 μm and a transition dipole–dipole interaction strength of = 27 μm and a transition dipole–dipole interaction strength of *V*/*h* ≈ 1 Hz. The latter value was estimated from the two-atom model given in ref. 47. For comparison, the natural line width of the Rb D lines is 6 MHz,^46^ the Doppler broadening in the atom beam is 560 MHz, and the self-broadening is 1 Hz.^50^ Hence, it would be extremely difficult to extract the dipolar interaction from line shape or dephasing properties, even at ultra low ensemble temperatures. As such, our measurement scheme provides an ideal and unique approach to study the long-range behavior of interparticle interactions.

## 4 Discussion

To ensure the reliability of our data, conceptual and experimental ambiguities have been thoroughly excluded. In a theoretical work, it has been pointed out, that by experimentally select the *n*QC signal components, one may reduce the experimental observable to an effective many-body operator due to which the higher-order signals arise from the single-atom response of the system as an intrinsic effect of the detection.^48^ Furthermore, accumulation effects of excited state populations due to decay rates being smaller or in the order of the laser repetition rate can produce artificial higher-order signals^51^ as well as nonlinearities in the detectors or detection electronics. All effects rely on higher-order signal generation from the single-atom 1QC interference signal. By depleting the 1QC signal with perpendicular pump–probe laser polarization, we can clearly exclude these ambiguities from contributing to our data. In addition, we note, that a laser repetition rate of 200 kHz is used in the current study, which is much smaller than the decay rate of excited Rb populations (6 MHz).

For strongly interacting systems, it has been pointed out that partial quenching of the two-photon fluorescence may occur, due to which the 2QC signal may not be a unique probe of interparticle interactions anymore.^52^ However, this effect is irrelevant at the low particle densities and interaction strengths of the here investigated systems. Moreover, fluorescence quenching is avoided in the photoelectron measurements which yield qualitatively the same results. Likewise, the influence of cooperative radiation phenomena, *e.g.* superradiance, cascading and local-field effects^53^,^54^ can be excluded by the low ensemble densities and photoionization measurements. Eventually, harmonic impurities in the phase modulation of pump and probe pulses or in the reference signal, used for the lock-in demodulation, appear outside of the detected frequency range in the Fourier spectra and thus do not contribute to the signal.

In general, with the perpendicularly polarized pump–probe measurements, we provide a generic argument for the presence of long-range interactions in the system which is based on simple considerations of *m*-level selection rules. Thereby, the experimental conditions and the discussed exclusion of other effects clearly point to transition dipole–dipole interactions as the underlying interaction mechanism.

To check the consistency with previous theoretical work, we also calculated the 1QC and 2QC signals for a minimalistic model and by adapting the perturbative treatment commonly used in nonlinear time-domain spectroscopy.^55^ Our model is similar to the one used in ref. 47 and basically consists of two isolated atoms that interact *via* transition dipole–dipole interactions. Each atom is approximated by a two-level system but in contrast to the previous study^47^ we now take magnetic sublevels into account. The atom's ground state is treated as s-type (no *m*-level), the excited state as p-type (*m*-level with *m* = –1, 0, 1), respectively. This results in a 3 × 3 interaction matrix,^56^,^57^ based on which we performed simulations in analogy to ref. 47. To accurately capture the effect of laser polarization, we included an average over all possible orientations of the dimer with respect to the pump and probe pulse polarizations. Our results show, that for perpendicular pump–probe polarization, the 1QC signal vanishes independent of the interaction strength. The 2QC collective signal disappears for non-interacting particles, independent of the laser polarization whereas with existing dipole–dipole interaction, the 2QC signal exists for parallel and also for perpendicular polarization. This is in accordance with the experiment.

For a quantitative comparison of signal magnitudes, extensive calculations using explicitly the level structure of Rb including hyperfine and magnetic sublevels would be necessary and an ensemble much greater than two atoms has to be considered. This will however greatly complicate the calculations and is beyond the scope of the current work. As such, we refrain from a detailed quantitative analysis of signal amplitudes in our data as a sufficiently elaborated theoretical model yet has to be developed.

Nonetheless, a comparison of the simplified two-atom model with the experimental data gives us a first hint on the relevance of many-body effects beyond two-body contributions. To this end, in Table 1, theoretical calculations are compared with the experimental results for both the single-atom two-photon resonance (5d) and the collective two-photon two-atom resonance (2D_2_). For calibration, both are normalized to the single-atom one-photon D_2_ resonance. The quantitative calculations are performed using the model of ref. 47, which beside being restricted to two atoms, also ignores *m*-levels. The calculation of the 5d intensity ratio is based on a single, three level system (5s,5p,5d). Dipole moments were taken from ref. 46 and 58. Uncertainties were estimated from uncertainties of experimental parameters used for the theoretical calculations. Note, that for the photoionization measurements, a comparison of the 5d resonance has been omitted since this would require accurate estimates of the photoionization probabilities, for which no literature values have been found for our specific ionization scheme.

**Table 1:** Comparison between experiment and a simple theoretical model which is restricted to two particles. Spectral amplitudes are given relative to the Rb D_2_ line amplitude

Density (cm^–3^)	Ratio (2D_2_/D_2_)		Ratio (5d/D_2_)	
	Exp.	Theo.	Exp.	Theo.
7 × 10^10^	2 × 10^–2^	2(2) × 10^–9^	1 × 10^–3^	2(2) × 10^–3^
8 × 10^9^	3 × 10^–3^	3(2) × 10^–10^	—	—
8 × 10^6^	2 × 10^–2^	6(12) × 10^–13^		

Interestingly, we find that the experimental two-atom signatures are more than seven orders of magnitude larger than predicted in our calculations. Clearly, this difference cannot be explained by the uncertainty in experimental parameters. Moreover, the single-atom two-photon excitation signal (5d) matches with our calculations within a factor of three. The large discrepancy between experiment and theory found for the multi-atom signals thus provides strong indication, that the nearest neighbor approximation used in the model is insufficient even for atomic systems of extremely low density and implies that interaction networks of much larger than two atoms may form in the system. Indications for many-body effects in atomic gases extending beyond two-atom interactions have been reported before, however only at much higher particle densities (>10^13^ cm^–3^)^29^,^33^ or for much larger transition dipole moments^59^,^60^ where >2-body cooperative effects are more likely expected. However, we note, that a much more elaborate model than the one used here, has to be developed to elucidate the details of the many-body interaction mechanism. The current study merely indicates, that a nearest neighbor pair-wise interaction is insufficient to explain the experimental findings.

## 5 Conclusions

In this work, we demonstrate in a specialized femtosecond quantum beat experiment the preparation and detection of delocalized excitons (delocalized over two and three atoms) in extremely dilute thermal atomic ensembles. Remarkably, we observe the many-body states down to particle densities of only 8 × 10^6^ cm^–3^, corresponding to a mean interatomic distance of , corresponding to a mean interatomic distance of 〈*r*〉 > 10 μm. This is surprising, as the mean atom–atom distance is much greater than the wavelength of the excitation field (∼790 nm) and collectively induced coherences should destructively interfere over the ensemble average. The clear signatures of excitonic states thus point to a significant long-range interaction in the system, presumably mediated by transition dipole fields, which have been so far considered to be negligibly small at the tested conditions. To confirm this, we employed perpendicularly polarized pulse sequences, which select the two-atom signals as a unique probe for the presence of interparticle interactions in the system. Yet, a quantitative comparison of theory and experiment yields a discrepancy of several orders of magnitude, implying, that fundamental many-body systems, such as atomic gases, still elude from an accurate description and more refined models are necessary to capture the physics of such systems. > 10 μm. This is surprising, as the mean atom–atom distance is much greater than the wavelength of the excitation field (∼790 nm) and collectively induced coherences should destructively interfere over the ensemble average. The clear signatures of excitonic states thus point to a significant long-range interaction in the system, presumably mediated by transition dipole fields, which have been so far considered to be negligibly small at the tested conditions. To confirm this, we employed perpendicularly polarized pulse sequences, which select the two-atom signals as a unique probe for the presence of interparticle interactions in the system. Yet, a quantitative comparison of theory and experiment yields a discrepancy of several orders of magnitude, implying, that fundamental many-body systems, such as atomic gases, still elude from an accurate description and more refined models are necessary to capture the physics of such systems.

In general, the presented work opens new possibilities for studies of interaction phenomena at extreme regions of phase space to test fundamental principles, *e.g.* Dicke physics or new phase transitions.^25^,^61^,^62^ In principle, our high sensitivity approach could be applied to isolated systems of single- to few-body character to enable precision experiments. Moreover, we have recently demonstrated that the method works also at low sampling/signal rates,^63^ which makes the approach compatible with ultra cold targets such as magneto-optical-, dipole-, or ion traps.

## Conflicts of interest

There are no conflicts to declare.
